# Widely Targeted Metabolomics Reveals Metabolite Diversity in Jalapeño and Serrano Chile Peppers (*Capsicum annuum* L.)

**DOI:** 10.3390/metabo13020288

**Published:** 2023-02-16

**Authors:** Dennis N. Lozada, Sahithi Reddy Pulicherla, Francisco Omar Holguin

**Affiliations:** 1 Department of Plant and Environmental Sciences, New Mexico State University, Las Cruces, NM 88003, USA; 2 Chile Pepper Institute, Department of Plant and Environmental Sciences, New Mexico State University, Las Cruces, NM 88003, USA; 3 Molecular Biology Interdisciplinary Life Sciences Program, Department of Entomology, Plant Pathology, and Weed Science, New Mexico State University, Las Cruces, NM 88003, USA

**Keywords:** flavonoids, human health, ‘NuMex LotaLutein’, ‘NuMex Pumpkin Spice’, nutrition, phenolic acids, ultra-performance liquid chromatography tandem mass spectrometry

## Abstract

Chile peppers (*Capsicum annuum* L.) are good sources of vitamins and minerals that can be included in the diet to mitigate nutritional deficiencies. Metabolomics examines the metabolites involved in biological pathways to understand the genes related to complex phenotypes such as the nutritional quality traits. The current study surveys the different metabolites present in jalapeño (‘NuMex Pumpkin Spice’) and serrano (‘NuMex LotaLutein’) type chile peppers grown in New Mexico using a widely targeted metabolomics approach, with the ‘NuMex LotaLutein’ as control. A total of 1088 different metabolites were detected, where 345 metabolites were differentially expressed; 203 (59%) were downregulated and 142 (41%) were upregulated (i.e., relative metabolite content is higher in ‘NuMex Pumpkin Spice’). The upregulated metabolites comprised mostly of phenolic acids (42), flavonoids (22), and organic acids (13). Analyses of principal component (PC) and orthogonal partial least squares demonstrated clustering based on cultivars, where at least 60% of variation was attributed to the first two PCs. Pathway annotation identified 89 metabolites which are involved in metabolic pathways and the biosynthesis of secondary metabolites. Altogether, metabolomics provided insights into the different metabolites present which can be targeted for breeding and selection towards the improvement of nutritional quality traits in *Capsicum*.

## 1. Introduction

The recent global health crisis necessitates the improvement of diets by consuming healthier and more nutritious foods. Chile peppers (*Capsicum* spp.) are good sources of vitamins and minerals that can mitigate nutritional deficiencies. These include phenols; ascorbic acid; carotenoids; chlorophylls; vitamins A, C, and E; capsaicinoids; and flavonoids [1,2]. Capsaicinoids, which give chile peppers their unique ‘heat’ sensation, possess anti-cancer properties [3] and have been shown to have the potential of alleviating cough, rheumatism, sore throat, and toothache [4]. Phenolic compounds show great potential as strong antioxidants that can aid the human body against free radicals resulting from reactive oxygen species (ROS) to help prevent cancer, cardiovascular disorders, and neurodegenerative diseases [5,6,7]. These metabolites have also demonstrated anti-aging and depigmentation properties, anti-inflammatory potential, and antimicrobial activity [8]. A survey on the variation of vitamin contents in a *Capsicum* germplasm identified eight cultivars that have higher vitamin A concentration than sweet potato (*Ipomea batatas*), and 16 pepper types that have vitamin C content higher than kiwi (*Actinidia deliciosa*), indicating a great potential for improving nutrition through genetic improvement [9].

A deeper understanding of the different metabolites present in chile peppers will provide avenues to understand the complex array of genes resulting in quantitative phenotypes such as the nutritional quality traits. Metabolomics studies link the phenotype and genotype, acting as a bridge between the phenome and the genome [10]. Previous estimates revealed at least 200,000 metabolites in the plant kingdom, significantly higher than those found in animals; a potential consequence of a wide variety of metabolic pathways developed by plants to thrive in diverse environmental conditions [11]. In chile peppers, metabolomics approaches have profiled metabolites involved in growth, development, and adaptation to different environments [12,13]. Numerous changes in the biochemical and physiological processes involving enzyme activity, gene expression, and metabolite synthesis result in developmental changes in the fruit in response to the environment [14]. For example, during fruit development in *C. chinense* Jacq, a metabolomics approach revealed distinct patterns of metabolite distribution at 16 days post-anthesis across the orange and red ripening periods [15]. In another study, fruit discoloration was attributed to the reduction in carotenoid content in discolored red peppers compared to normal peppers, where lipid and flavonoid synthesis were significantly associated with discoloration [16]. Increased lipid metabolism and decrease in capsaicinoid, flavones, flavanol compounds, and terpenoids were further observed in domesticated chile peppers compared to their wild ancestors [17], indicating that genetic improvement for increased nutritional quality through breeding and selection have been implemented for the cultivated chile peppers.

Widely targeted metabolomics is the next generation of metabolomics, combining the benefits of the targeted and untargeted approaches, and has several advantages, including being high-throughput, highly sensitive, and having a broad coverage [18], in contrast to targeted approaches where some metabolites may be left unidentified [19]. The detection and identification of broadly targeted metabolites are made possible by Q-TRAP mass spectrometry based on multiple reaction monitoring mode, which allows for the simultaneous quantification of hundreds of known metabolites over a thousand of unknown metabolites [20,21].

The objectives of this study were to: (1) survey the metabolite diversity in jalapeño and serrano peppers using a nontargeted metabolomics approach; (2) identify differentially expressed metabolites; and (3) perform the functional annotation of genes involved in different metabolic pathways. A widely targeted metabolomics approach through an ultra-performance liquid chromatography tandem mass spectrometry (UPLC-MS/MS) was employed to determine differentially expressed metabolites in jalapeño and serrano pepper types cultivated in a New Mexico growing environment. The results from this study will be relevant for future metabolite-based genomics-assisted mapping approaches for the genetic improvement of nutritional quality traits in *Capsicum*.

## 2. Materials and Methods

### 2.1. Plant Material and Collection of Fruit Samples

**Figure 1:**
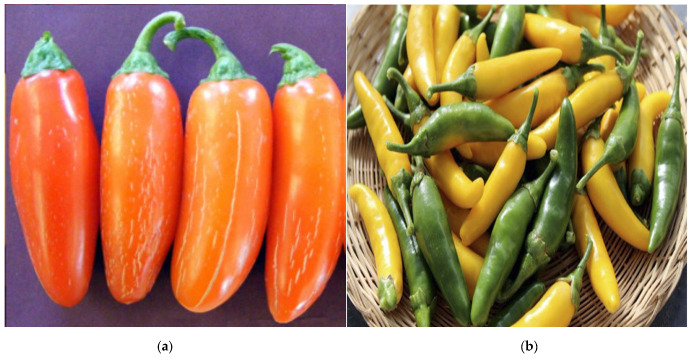
‘Numex Pumpkin Spice’ (**a**) and ‘NuMex LotaLutein’ (**b**) fruits showing their characteristic orange and yellow colors, respectively. ‘NuMex Pumpkin Spice’ is a colored jalapeño, whereas ‘NuMex LotaLutein’ is a biofortified serrano type with improved lutein content. Adapted with permission from Refs. [22,23]. ©2015, 2020, American Society for Horticultural Science.

Two *C. annuum* L. cultivars previously released by the New Mexico State University (NMSU) Chile Pepper Breeding and Genetics Program, viz., ‘NuMex Pumpkin Spice’ (a jalapeño type) [22] and ‘NuMex LotaLutein’ (a serrano type), [23] were used in this study (Figure 1). The ‘NuMex Pumpkin Spice’ originated from the hybridization between the ‘Permagreen’ bell pepper and ‘Early Jalapeno’ and was developed using a pedigree breeding approach with three generations of backcrossing to a jalapeño fruit type, followed by multiple cycles of single plant selections [22]. ‘NuMex LotaLutein’ is a biofortified serrano pepper with improved lutein content and was derived from a segregating F_2_ population resulting from selfing a commercial F_1_ hybrid serrano cultivar [23]. ‘NuMex Pumpkin Spice’ has a characteristic deep orange (i.e., ‘pumpkin’-like) color, whereas ‘NuMex LotaLutein’ has a distinct yellow color.

The cultivars were transplanted at the Amy Goldman Flower Chile Pepper Institute Teaching Garden, Fabián García Science Center, Las Cruces, NM, USA. Three mature fruits from three individual plants (representing three biological replicates) from each cultivar were sampled ~130 days after transplanting (~190 days after initial sowing in the greenhouse). Fruit samples were subsequently freeze-dried for ~48 h using a Labconco Freeze Dry System (Labconco Corporation, Kansas City, MO, USA) and were sent to MetWare^®^ Bio, MA, USA (https://www.metwarebio.com/ (accessed on 4 November 2022)) for processing and metabolomics analyses.

### 2.2. UPLC-MS/MS Based Widely Targeted Metabolomics Approach

Biological samples were re-freeze-dried using a vacuum lyophilizer (Scientz-100 F) at MetWare^®^ Bio. A mixer mill (MM 400, Retsch, Verder Scientific, Inc., Newtown, PA, USA) with zirconia beads was used to grind the samples for 1.5 min at 30 Hz. The freeze-dried powder samples (~50 mg) were dissolved with 70% methanol solution (1.2 mL; 0.84 mL methanol; 0.36 mL distilled water), mixed using a vortex for 30 sec every 30 min, and stored at 4 °C overnight. The extracts were later centrifuged at 12,000 rpm for 10 min and filtered using a 0.22 μm filter before liquid chromatography and mass spectrometry.

Ultra-performance liquid chromatography (UPLC) (ExionLC™ AD, https://sciex.com.cn/ (accessed on 4 November 2022)) and tandem mass spectrometry (MS/MS) (Applied Biosystems QTRAP 6500, https://sciex.com.cn/ (accessed on 4 November 2022)) were performed with the following conditions (a): liquid phase chromatographic column: Agilent SB-C18 1.8 µm, 2.1 mm × 100 mm; (b) mobile phase: A phase—ultrapure water (0.1% formic acid added), B phase—acetonitrile (0.1% formic acid added); (c) elution gradient: 0.00 min, the proportion of B phase was 5%, within 9.00 min, the proportion of B phase increased linearly to 95% and remained at 95% for 1 min, 10.00–11.10 min, the proportion of B phase decreased to 5% and balanced at 5% up to 14 min; (d) flow rate: 0.35 mL/min; (e) column temperature: 40 °C; and (f) injection volume: 2 μL.

Mass spectrum scans were acquired using both the Linear Ion Trap (LIT) and Triple Quadrupole (QqQ) modes of a hybrid QqQ LIT Mass Spectrometer (Q TRAP ^®^) (AB6500 Q TRAP^®^ UPLC/MS/MS system) with an ESI Turbo ion spray interface, and both cation and anion modes were controlled by Analyst 1.6.3 software (AB Sciex, MA, USA). Operating conditions consisted of the following: turbine spray (ion source); source temperature 550 °C; ion spray voltage (IS) 5500 V (cation mode)/−4500 V (anion mode); ion source gas I (GSI), gas II (GSII), and curtain gas (CUR) set to 50, 60, and 25 psi, respectively; collision-induced ionization parameter set to high. The multiple reaction monitoring (MRM) mode was used for the QqQ scan, where a specific set of MRM ion pairs was monitored based on the eluted metabolites at each period.

### 2.3. Qualitative and Quantitative Analyses of Metabolites

An in-house database (MetWare^®^ Database) was used to identify metabolites based on their secondary spectrum information. The following were excluded during the qualitative analyses: signals from isotopes, and repeated signals containing cations such as K^+^, Na^+^, and NH_4_^+^. Semi-quantitative analyses of the samples were performed by MRM using QqQ MS. Peak integration on the mass spectrum peaks of all the metabolites identified was performed after obtaining spectrum analyses data from the different samples. Integral correction was implemented on the peaks of similar metabolites in different samples according to Fraga et al. [24].

### 2.4. Sample Quality Control and Correlation Analysis

A quality control (QC) sample was prepared from a mixture of sample extracts to examine the repeatability of the sample under the same treatment conditions. Metabolite extraction and detection repeatability were evaluated by analyzing the overlapping display of total ion chromatogram (TIC) of different QC samples. The high stability of the instrument was related to the high repeatability and reliability of the data. The correlation between samples was established using the ‘cor’ function in R software (www.r-project.org (accessed on 4 November 2022)).

### 2.5. Analysis of Principal Components and Discriminant Analysis by Orthogonal Partial Least Squares

Metabolite data were compressed into *N* principal components (PCs) to describe the features of the dataset, where the first principal component (PC1) represented the highest degree of variation, followed by the second principal component (PC2), and so on. Principal components analysis (PCA) was performed using the ‘prcomp’ function in the R program, where parameter scale = ‘True’ demonstrated the unit variance (UV) scaling for normalizing the data. Biplots were constructed using the first two PCs (PC1 and PC2). Orthogonal partial least squares discriminant analysis (OPLS-DA) for the metabolome data was further implemented to show the differences between each group. During the modeling of OPLS-DA, ***X*** matrix information is divided into information that is either related or unrelated (i.e., noise) to ***Y***, where the variables associated with ***Y*** are the PC, and the information unrelated to ***Y*** is the orthogonal PC [25]. The ‘OPLSR.Anal’ function in the package ‘MetaboAnalystR’ in R was used for OPLS-DA. S plots for the OPLS-DA were constructed by plotting the correlation coefficient values between the PCs and the metabolites against the covariance between the PCs and metabolites. The OPLS-DA model validation was performed using permutation tests, represented as *R*^2^*X*, *R*^2^*Y*, and *Q*^2^, where *R*^2^*X* and *R*^2^*Y* denote the explanatory rate of the model to the ***X*** and ***Y*** matrices, respectively; and *Q*^2^ signifies the prediction ability of the model. Values closer to 1 indicate a more stable and reliable model for validation.

### 2.6. Identification and Functional Annotation of Differentially Expressed Metabolites

Variable importance in projection (VIP) from modeling using OPLS-DA was used to determine metabolites that were differentially expressed. Given that the number of biological replicates, *n*, is ≥3, the different metabolites were screened by combining the fold change and VIP of the discriminant analysis model, where metabolites with VIP ≥ 1 and with fold change ≥2 or ≤0.5 at *p* < 0.05 were considered to be differentially expressed. Volcanic plots of the differentially expressed metabolites were constructed by plotting VIP values against the log_2_ (fold change; FC). Functional annotation of the differential metabolites was performed using the KEGG (Kyoto Encyclopedia of Genes and Genomes) database [26] based on the KO (KEGG Orthology) of molecular functions. The PCA, OPLS-DA model validations, and functional annotation of the different metabolites were all performed at MetWare^®^ Bio. Screening results were based on performing comparisons of metabolite contents where the ‘NuMex LotaLutein’ was used as the control. Positive and negative values for log_2_FC indicate upregulation and downregulation, respectively. Upregulation signifies that the relative content for the metabolite is higher in ‘NuMex Pumpkin Spice’ than in ‘NuMex LotaLutein’ and vice versa.

## 3. Results

### 3.1. Fruit Sample Collection and Metabolite Identification

**Figure 2:**
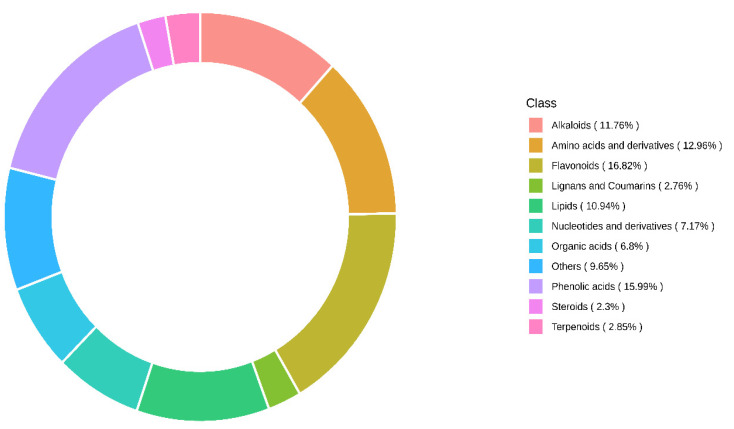
Proportion of different classes of compounds for the metabolites detected from jalapeño and serrano chile peppers using a widely targeted metabolomics approach.

Mature fruit samples of ‘NuMex LotaLutein’ and ‘NuMex Pumpkin Spice’ were collected for metabolomic profiling. After the initial freeze drying, the weight of the ‘NuMex LotaLutein’ samples ranged between 1.72 and 2.77 g, whereas for ‘NuMex Pumpkin Spice’ weights ranged between 1.28 and 2.35 g. Using a UPLC-MS/MS system and the MetWare^®^ in-house database, a total of 1088 metabolites were identified. Flavonoids and phenolic acids comprised 16.82% and 15.99% of the identified metabolites, respectively. Along with them, amino acids and derivatives (12.96%), alkaloids (11.76%), and lipids (10.94%) were also present in considerable quantities (Figure 2).

### 3.2. Analysis of Principal Components and Orthogonal Partial Least Squares

**Figure 3:**
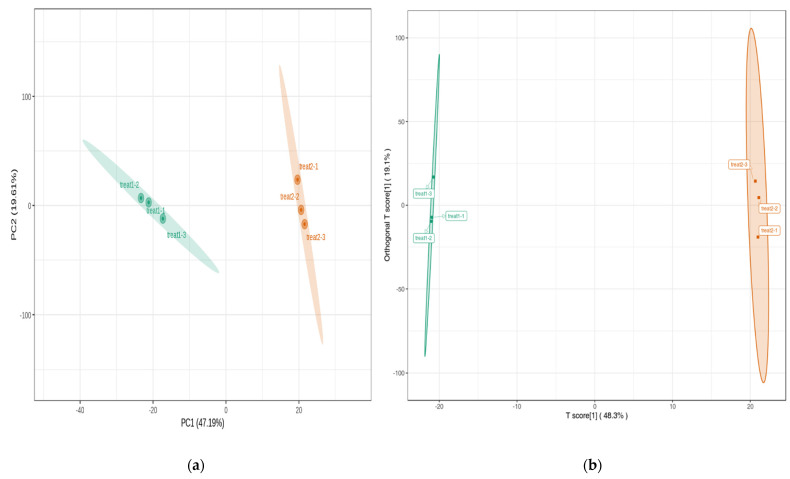
(**a**) Principal component (PC) biplot for the PC1 and PC2 reveal the overall variation between the cultivars in terms of metabolomic profiles. (**b**) Orthogonal partial least squares discriminant plot for the chile pepper cultivars. Treatment 1: ‘NuMex LotaLutein’; treatment 2: ‘NuMex Pumpkin Spice’. Numbers 1, 2, and 3 correspond to the first, second, and third biological replications, respectively.

**Figure 4:**
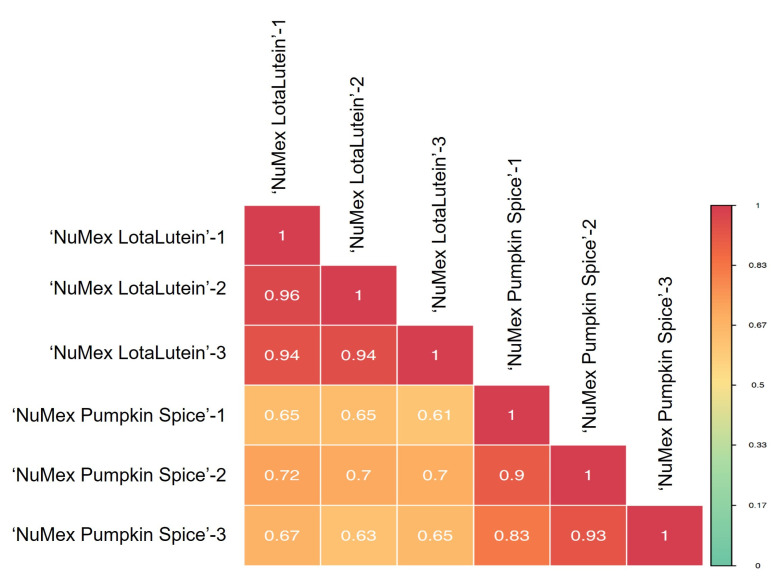
Pearson correlation coefficients among the biological replicates for the cultivars. Numbers 1, 2, and 3 correspond to the first, second, and third biological replications, respectively. Correlation coefficients were significant at *p* < 0.0001.

PCA has identified two distinct groups based on the cultivars, where the biological replicates from each sample were grouped together (Figure 3a). Group 1, which consisted of the ‘NuMex LotaLutein’ samples, clustered on the left side, whereas Group 2, comprising ‘NuMex Pumpkin Spice’ samples, clustered on the right side of the PCA diagram. The first principal component (PC1) contributed to 47.19% of variation, whereas PC2 was associated with 15.95% of variation. Model prediction by OPLS-DA modeling revealed consistent clustering for the samples according to cultivars (Figure 3b). A significant model for partial LS discriminant analyses was supported by the high values of the explanatory rates, *R*^2^*X* (0.67), *R*^2^*Y* (1.0), and of the prediction ability of the model, *Q*^2^ (0.94). Excluding the QC samples, the overall contributions of the T-score and orthogonal T-score for the OPLS-DA were 48.3 and 19.1%, respectively. The results from correlation analyses further supported the differentiation observed from PCA. Mean Pearson correlation coefficients of 0.92 were observed within cultivars, whereas an average of 0.66 was observed across treatments (Figure 4). Coefficient of variation (CV) values across all the QC samples also demonstrated the stability of the experimental data, with the proportion of samples having a CV less than 0.3 and 0.5 being higher than 75% and 85%, respectively. The CV across all metabolites ranged between 1.34% and 115.54%, with a mean value of 36.50%.

### 3.3. Identification and Functional Annotation of Differentially Expressed Metabolites

A total of 345 metabolites (32%) were differentially expressed, where 203 (59%) of these were downregulated and 142 (41%) were upregulated (Table S1). The upregulated metabolites were classified into 10 different classes of compounds, which mostly comprised phenolic acids (42; 30%), flavonoids (22; 15%), organic acids (13; 9%), and amino acids and derivatives (11; 8%). The downregulated metabolites mainly consisted of flavonoids (43; 21%) phenolic acids (41; 20%), and alkaloids (32; 16%), and were grouped into nine different classes. Values for log_2_FC of the upregulated metabolites ranged between 1 and 18, with a mean value of 2.59. An average VIP value of 1.31 was observed for all the upregulated metabolites. Capsaicin and related compounds were not differentially expressed.

Among the top 10 upregulated metabolites, eight were classified as phenolic acids, and two were flavonoids (Table 1). The average VIP and log_2_FC values for the top 10 upregulated metabolites were 1.42 and 9.52, respectively. 1-Phenylethanol, Dicaffeoylshikimic acid, 2-Methoxycinnamic acid, 1,3-O-Dicaffeoylquinic Acid (Cynarin), and 4-O-(4′-O-alpha-D-Glucopyranosyl)caffeoylquinic acid, all phenolic acids, were the top five upregulated metabolites, with log_2_FC values of 18.04, 16.4, 13.33, 8.06, and 7.52, respectively. For the top ten downregulated metabolites, three were phenolic acids, six were flavonoids, and one was an alkaloid. Mean values for the VIP and log_2_FC for the top 10 downregulated metabolites were 1.42 and -91.68, respectively. Phenolic acids and flavonoids comprised the top five downregulated metabolites; namely, 3,6-Di-O-caffeoyl glucose (log_2_FC value = −16.73), Rosmarinic acid methyl ester (−15.36), 2,3-Dimethoxybenzaldehyde(−14.93) (all three are phenolic acids), and Quercetin-3-O-(2′′-O-rhamnosyl)rutinoside-7-O-glucoside (−11.96) and Quercetin-3-O-(6′′-O-acetyl)glucosyl-(1→3)-Galactoside (−6.01) (both flavonoids).

**Figure 5:**
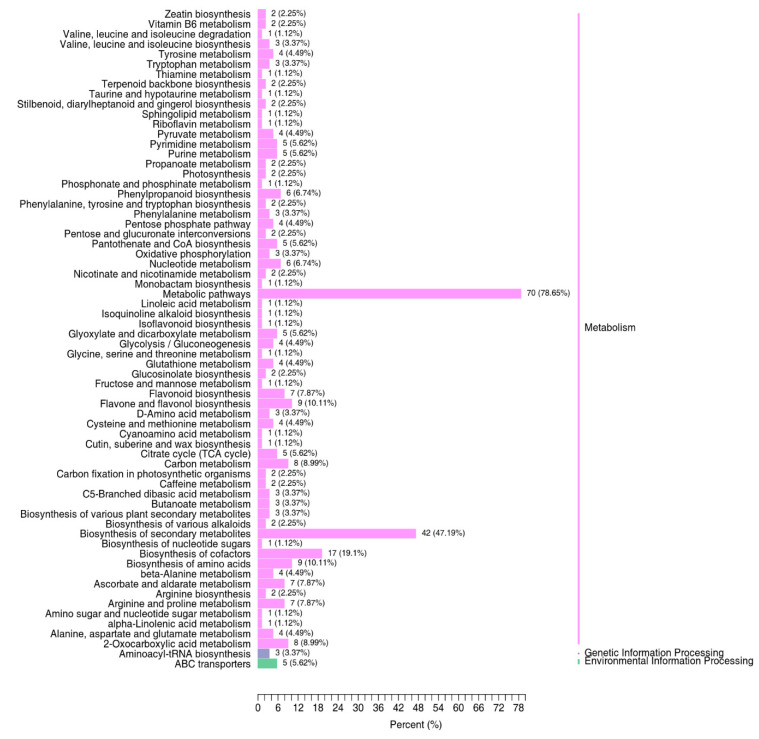
Summary of the different functions annotated for the differentially expressed metabolites (DEMs) using the KEGG database. Annotations revealed that the majority of the DEMs are involved in metabolic pathways and in the biosynthesis of secondary metabolites and cofactors. Note: A DEM can have multiple annotated functions.

**Figure 6:**
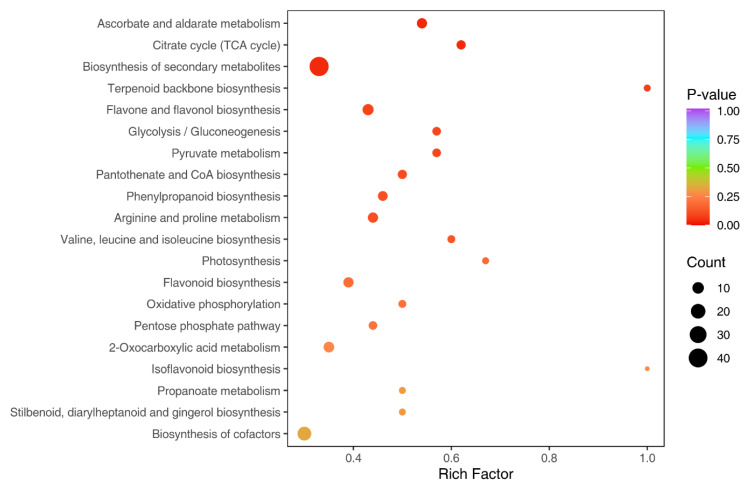
Rich factor (RF) values for the differentially expressed metabolites with annotated functions on the KEGG database. RF is the ratio of the number of differentially expressed metabolites in a pathway to the total number of metabolites annotated in the same pathway (RF for a pathway = no. of differentially expressed metabolites/total number of metabolites). Terpenoid backbone biosynthesis had significant enrichments, with an RF value of 1.

The KEGG database was used in order to understand the metabolite content as a whole network in the form of metabolic pathways. A total of 89 differentially expressed metabolites had annotation from the KEGG database, where 36 metabolites were upregulated and 53 were downregulated (Table S2). The top 10 upregulated metabolites comprised mostly phenolic acids, whereas the top 10 downregulated metabolites with annotation consisted mostly of flavonoids (Table 2). Classification based on annotation from the KEGG database revealed that of the differentially expressed metabolites, 70 (78.65%) were annotated to be involved in metabolic pathways. A total of 42 (47.19%) and 17 (19.1%) metabolites were associated with the biosynthesis of secondary metabolites and of cofactors, respectively (Figure 5). The upregulated metabolites with KEGG annotation were classified into seven different groups that comprised organic acids, nucleotides and derivatives, and flavonoids, among others. Similarly, downregulated metabolites comprised eight different groups, including amino acids and derivatives, organic acids, alkaloids, and phenolic acids. Flavonoids, phenolic acids, organic acids, and nucleotides and derivatives comprised the top 10 differentially expressed upregulated metabolites with KEGG database map annotation. The significant enrichment of metabolites involved in terpenoid backbone biosynthesis was demonstrated (rich factor (RF) value of 1). Differential metabolites involved in photosynthesis also showed significant enrichment, with an RF value equal to 0.65 (Figure 6). Functions related to the biosynthesis of secondary metabolites including lutein (β,ε-carotene-3,3′-diol), the predominant compound in the ‘NuMex LotaLutein’ serrano pepper, have been annotated in the KEGG Orthology database for a number of differentially expressed metabolites identified.

## 4. Discussion

Chile pepper fruits possess many compounds of high nutritive values which can be integrated in the human diet to mitigate nutritional deficiencies. Crop nutrition is important not only for offering quality and economic value to growers, but also for providing nutritional security to consumers [27]. Since their initial application to examine plant functional genomics in the early 2000s [28,29,30], metabolomics approaches have been used to understand the different biochemical and physiological pathways for the selection of optimal alleles for the improvement of crops [31]. In the current study, a representative jalapeño and a serrano cultivar grown in New Mexico, USA, were subjected to widely targeted metabolomics to survey the different metabolites present in their fruit samples. The identified metabolites were able to distinguish the representative cultivars. The majority of the differentially expressed metabolites comprised the phenolic acids and the flavonoids. Metabolomics could be integrated with genomics-assisted approaches to facilitate the breeding and selection of chile pepper cultivars with improved nutritive value for consumers.

### 4.1. Metabolite Profiles Successfully Discriminate Jalapeño and Serrano Type Chile Peppers

Though crop metabolite profiling is an important area of nutritional and health research, this information can also be used for the classification of peppers. Principal components and partial least discriminant analyses are relevant for the classification of metabolomic datasets based on different factors such as variety, location, and their interactions [32]. In the current study, the jalapeño and serrano type chile peppers were differentiated based on their metabolite profiles. Both PCA and OPLS-DA showed consistent clustering based on cultivars, with total variation explained by the first two PCs reaching a total of 63%, indicating that the cultivars were significantly different from each other. The values for *Q*^2^, *R*^2^*X*, and *R*^2^*Y* derived from performing OPLS-DA further support the good predictive ability and reproducibility of the model in classifying the cultivars based on metabolomic information. Correlation analyses within sample treatments were significant (*p* < 0.0001), demonstrating clustering based on the cultivars. Genomic information using 66,650 single nucleotide polymorphism markers, nonetheless, previously classified ‘NuMex LotaLutein’ and ‘NuMex Pumpkin Spice’ in the same cluster [33], indicating high genetic relatedness; yet, implementing a metabolomic approach can resolve differences between the cultivars.

Metabolite profiles have been previously used for species differentiation and cultivar identification in chile peppers. Capsaicinoid and flavonoid contents, for example, were recently used as biochemical markers to distinguish Jize from Korean chile peppers [34]. In another study, an untargeted metabolomics approach identified biomarkers, including flavonoids, steroids, terpenoids, anthocyanin, and carboxylic acids, and characterized three *Capsicum* species (*C. baccatum, C. chinense*, and *C. frutescens*) from a collection of native South American chile peppers [35]. Eleven Mexican *C. annuum* cultivars were differentiated based on their metabolomic fingerprints, which consisted of sugars, amino acids, organic acids, polyphenolic acids, and alcohols [36]. Serrano peppers from Mexico were grouped in two different clusters based on their metabolite profiles, which included organic acids, sugars, amino acids, and polyphenols [37]. In contrast, using a single metabolite (i.e., capsaicin) alone did not successfully discriminate ‘Superhot’ chile peppers cultivated across multi-year trials in New Mexico [38]. Taken together, our results and those of previous studies indicate that a ‘macroscale’ metabolomic profiling would be more advantageous than using only a single metabolite in cultivar identification and species differentiation in *Capsicum*.

### 4.2. Differentially Expressed Metabolites Have Implications on Human Health and Nutrition

Widely targeted metabolomics for jalapeño and serrano peppers revealed differentially expressed metabolites comprising mostly of upregulated phenolic acids and flavonoids. Phenolic acids are among the most commonly found compounds in *Capsicum* fruits and have been observed to possess vital functions in human health, nutrition, and the prevention of diseases because of their antioxidant, antiviral, and antibacterial properties [5,39]. Phenolics have been previously identified in different species of peppers, including *C. annuum* cultivars from India [40]; *C. annuum*, *C. chinense*, and *C. baccatum* cultivated in New Mexico [41]; *C. baccatum* [42]; and *C. frutescens* and *C. chinense* cultivars from Peru, South America [35]. The human body cannot produce phenolic compounds; hence, they must be acquired from food sources [5]. Major phenolic acid derivatives, caffeic acid (CA), sinapic acid (SA), and ferulic acid (FA) [5], were observed to be differentially expressed in the present work; CA and FA were upregulated, whereas SA was downregulated. Flavonoids (e.g., quercetin and luteolin) were also detected to be differentially expressed, consistent with previous studies [43,44,45,46]. A total of 23 quercetin metabolites were found to be differentially expressed, where five metabolites (namely, quercetin-3-O-sambubioside-5-O-glucoside, 3-O-methylquercetin, 3,7-di-O-methylquercetin, quercetin-3-O-(2′′-O-malonyl)sophoroside-7-O-arabinoside, and 6-methoxyquercetin-3-O-rhamnoside) were upregulated and the remaining 18 metabolites were downregulated. A total of 10 differentially expressed metabolites belong to the luteolin class, comprising mostly upregulated compounds (luteolin-4′-O-glucoside, luteolin-7-O-glucoside (cynaroside), luteolin-7-O-sophoroside-5-O-arabinoside, luteolin-7-O-gentiobioside, luteolin-7,3′-di-O-glucoside, and luteolin-7-O-glucuronide). Capsaicin and related families of ‘pungent’ alkaloids (or ‘pseudoalkaloids’), however, were not observed to be differentially expressed in the current study, a potential consequence of the maturity (age) of the fruit samples used for analyses. Overall, the information for the differentially expressed metabolites and their corresponding chemical classes reveal that pepper fruits can be good sources of these nutritional compounds in the diet.

The KEGG map annotations for a number of the differentially expressed metabolites were related to multiple metabolic pathways such as the biosynthesis of secondary metabolites (KO01110), including lutein (β,ε-carotene-3,3′-diol), which is the predominant metabolite in the ‘NuMex LotaLutein’ serrano pepper [23]. The production of lutein in plants generally takes place through the mevalonate pathway, in which isopentyl diphosphate (IPP) is converted to geranylgeranyl pyrophosphate (GPP), which is then synthesized into phytoene and lycopene, the precursor of lutein [47,48,49]. The most upregulated phenolic acid, chlorogenic acid (3-O-caffeoylquinic acid), had four different pathway annotations related to phenylpropanoid biosynthesis (pathway ID: KO00940); flavonoid biosynthesis (KO00941); stilbenoid, diarylheptanoid, and gingerol biosynthesis (KO00945); and the biosynthesis of secondary metabolites (KO01110). 3,7-Di-O-methylquercetin (flavonoid) and phenol (phenolic acid) had two pathway annotations; namely, K00944 and KO01110 (secondary metabolite biosynthesis), and KO00350 (Tyrosine metabolism) and KO01110 (secondary metabolite biosynthesis). Adenosine 5′-triphosphate (ATP), also an upregulated metabolite, has seven different KEGG pathway annotations, including oxidative phosphorylation (KO00190), photosynthesis (KO00195), nucleotide metabolism (KO01232), and the biosynthesis of cofactors (KO01240), among others. Altogether, these functional annotations indicate the relevance of these upregulated compounds in the production of a wide array of important nutritional metabolites in *Capsicum*.

### 4.3. Integrating Metabolomics with Genomics Can Direct Breeding and Selection Decisions for Nutritional Quality Trait Improvement in Chile Pepper

Metabolomics can render insights into the different metabolites which can be targeted further for breeding and selection using different genomics tools such as metabolite-quantitative trait loci (mQTL) analyses, metabolite-based genome-wide association studies (mGWAS), and metabolite-genome predictions [50]. Variation in the phenolic acid content in chile peppers is affected by the varietal type, location, and growing season, and, hence, can impose challenges on performing breeding and selection [32]. Given the complex nature of these phenotypes, caution must be observed when performing the collection of fruit samples for metabolomics analyses to minimize the errors and variation due to sampling. The high accuracy, reliability, and stability of the widely targeted metabolomics approach implemented in the current study, as indicated by the high values of *R*^2^*X*, *R*^2^*Y*, and model prediction ability, and the low values for CV, nonetheless, can mitigate the effects of sampling and environmental variation. It might also be necessary to survey the metabolite compositions of cultivated and wild relatives of the chile pepper for a more robust foundation for targeted genomic breeding strategies [39]. Altogether, ‘multi-omics’ tools (e.g., genomics + metabolomics) can help plant breeders perform informed breeding and selection decisions for the development of cultivars with improved nutritional quality traits [51].

## 5. Conclusions

A widely targeted metabolomics approach revealed differentially expressed metabolites in jalapeño (‘NuMex Pumpkin Spice’) and serrano (‘NuMex LotaLutein’) type peppers grown in New Mexico. Metabolomic profiles successfully distinguished the cultivars, indicating that metabolites can serve as efficient markers to differentiate chile pepper varieties. Upregulated metabolites comprised of phenolic compounds and flavonoids, which have potential implications in improving human health and nutrition when incorporated in the diet. The diversity of metabolites observed in the present work supports the nutritional value of chile peppers and can direct important breeding and selection decisions for designing more nutritious specialty vegetable crops in the future.

## Figures and Tables

**Table 1:** Top 10 most highly upregulated and downregulated metabolites.

Metabolite	Class	VIP ^1^	Fold Change (FC)	log_2_FC ^2^
1-Phenylethanol	Phenolic acids	1.44	269,754.80	18.04
Dicaffeoylshikimic acid	Phenolic acids	1.44	86,594.42	16.40
2-Methoxycinnamic acid	Phenolic acids	1.44	10,264.53	13.33
1,3-O-Dicaffeoylquinic Acid (Cynarin)	Phenolic acids	1.42	266.71	8.06
4-O-(4′-O-alpha-D-Glucopyranosyl)caffeoylquinic acid	Phenolic acids	1.43	182.93	7.52
Chlorogenic acid methyl ester	Phenolic acids	1.44	103.94	6.70
Cyanidin-3-O-(6′′-O-caffeoyl-2′′-O-xylosyl)glucoside	Flavonoids	1.43	82.45	6.37
Tri-O-galloyl Methyl gallate	Phenolic acids	1.41	82.20	6.36
Luteolin-7-O-Sophoroside-5-O-arabinoside	Flavonoids	1.43	74.07	6.21
5-O-Feruloyl quinic acid glucoside	Phenolic acids	1.37	73.70	6.20
3,6-Di-O-caffeoyl glucose	Phenolic acids	1.44	9.18 × 10^−6^	−16.73
Rosmarinic acid methyl ester	Phenolic acids	1.44	2.37 × 10^−5^	−15.36
2,3-Dimethoxybenzaldehyde	Phenolic acids	1.44	3.2 × 10^−5^	−14.93
Quercetin-3-O-(2′′-O-rhamnosyl)rutinoside-7-O-glucoside	Flavonoids	1.44	2.5 × 10^−4^	−11.96
Quercetin-3-O-(6′′-O-acetyl)glucosyl-(1→3)-Galactoside	Flavonoids	1.41	0.016	−6.01
p-Coumaroylagmatine	Alkaloids	1.43	0.020	−5.64
Quercetin-7-O-(6′′-malonyl)glucosyl-5-O-glucoside	Flavonoids	1.42	0.023	−5.43
Quercetin-3-O-rutinoside-7-O-rhamnoside	Flavonoids	1.40	0.0251	−5.31
Quercetin-3-O-xylosyl(1→2)glucosyl(1→2)glucoside	Flavonoids	1.40	0.0253	−5.30
Quercetin-3-O-(6′′-O-malonyl)glucosyl-5-O-glucoside	Flavonoids	1.38	0.031	−5.00

^1^ VIP—Variable importance in projection value. ^2^ Positive and negative values for log_2_FC indicate upregulation and downregulation, respectively. Screening results were based on a comparison where ‘NuMex LotaLutein’ was used as the control. Upregulation signifies that the relative content of the metabolite is higher in ‘NuMex Pumpkin Spice’ than in ‘NuMex LotaLutein’ and vice versa.

**Table 2:** Top 10 differentially expressed upregulated metabolites with KEGG database annotation.

Metabolite	Class	Variable Importance in Projection (VIP) Value	Fold Change (FC)	log_2_FC	KEGG Orthology (KO) Index	Annotation
Chlorogenic acid (3-O-Caffeoylquinic acid)	Phenolic acids	1.43	20.46	4.35	KO00940; KO00941; KO00945; KO01110	Phenylpropanoid biosynthesis; flavonoid biosynthesis; stilbenoid, diarylheptanoid and gingerol biosynthesis; metabolic pathways
3,7-Di-O-methylquercetin	Flavonoids	1.34	11.21	3.49	KO00944; KO01110	Flavone and flavonol biosynthesis; metabolic pathways
Phenol	Phenolic acids	1.42	11.01	3.46	KO00350; KO01100	Tyrosine metabolism; metabolic pathways
ATP; Adenosine 5′-Triphosphate	Nucleotides and derivatives	1.42	8.39	3.07	KO00190; KO00195; KO00230; KO00908 KO01100; KO01110; KO01232; KO01240	Oxidative phosphorylation; photosynthesis; purine metabolism; zeatin biosynthesis; metabolic pathways; nucleotide metabolism; biosynthesis of cofactors
2-Furoic acid	Organic acids	1.38	5.77	2.53	KO01100	Metabolic pathways
Kaempferol-3-O-rutinoside(Nicotiflorin)	Flavonoids	1.18	5.61	2.49	KO00944; KO01110	Flavone and flavonol biosynthesis; metabolic pathways
2-Oxoheptanedionic acid	Organic acids	1.37	5.55	2.47	KO01100; KO01210; KO01240	Metabolic pathways; 2-oxocarboxylic acid metabolism; biosynthesis of cofactors
Kaempferol-3-O-rhamnoside (Afzelin)(Kaempferin)	Flavonoids	1.31	4.92	2.30	KO00944	Flavone and flavonol biosynthesis
Luteolin-7-O-glucuronide	Flavonoids	1.26	4.76	2.25	KO00944	Flavone and flavonol biosynthesis
3-O-Methylquercetin	Flavonoids	1.40	4.56	2.19	KO00944; KO01110	Flavone and flavonol biosynthesis; metabolic pathways

## Data Availability

Data will be available from the authors upon reasonable request due to privacy or ethical restrictions.

## Supplementary Materials

The following supporting information can be downloaded at: https://www.mdpi.com/article/10.3390/metabo13020288/s1, Table S1: Metabolites identified for jalapeno- and serrano-type chile peppers, Table S2: Differentially expressed metabolites identified for jalapeno- and serrano-type chile peppers and their corresponding map annotations from the KEGG database.
